# Prediction and analysis of protein solubility using a novel scoring card method with dipeptide composition

**DOI:** 10.1186/1471-2105-13-S17-S3

**Published:** 2012-12-07

**Authors:** Hui-Ling Huang, Phasit Charoenkwan, Te-Fen Kao, Hua-Chin Lee, Fang-Lin Chang, Wen-Lin Huang, Shinn-Jang Ho, Li-Sun Shu, Wen-Liang Chen, Shinn-Ying Ho

**Affiliations:** 1 Department of Biological Science and Technology, National Chiao Tung University, Hsinchu, Taiwan; 2 Institute of Bioinformatics and Systems Biology, National Chiao Tung University, Hsinchu, Taiwan; 3 Department of Anesthesiology, Tri-Service General Hospital, Taipei, Taiwan; 4 Department of Multimedia Entertainment Science, Asia Pacific Institute of Creativity, Miaoli, Taiwan; 5 Department of Automation Engineering, National Formosa University, Yunlin, Taiwan; 6 Department of Information Management, Overseas Chinese University, Taichung Taiwan

## Abstract

**Background:**

Existing methods for predicting protein solubility on overexpression in *Escherichia coli *advance performance by using ensemble classifiers such as two-stage support vector machine (SVM) based classifiers and a number of feature types such as physicochemical properties, amino acid and dipeptide composition, accompanied with feature selection. It is desirable to develop a simple and easily interpretable method for predicting protein solubility, compared to existing complex SVM-based methods.

**Results:**

This study proposes a novel scoring card method (SCM) by using dipeptide composition only to estimate solubility scores of sequences for predicting protein solubility. SCM calculates the propensities of 400 individual dipeptides to be soluble using statistic discrimination between soluble and insoluble proteins of a training data set. Consequently, the propensity scores of all dipeptides are further optimized using an intelligent genetic algorithm. The solubility score of a sequence is determined by the weighted sum of all propensity scores and dipeptide composition. To evaluate SCM by performance comparisons, four data sets with different sizes and variation degrees of experimental conditions were used. The results show that the simple method SCM with interpretable propensities of dipeptides has promising performance, compared with existing SVM-based ensemble methods with a number of feature types. Furthermore, the propensities of dipeptides and solubility scores of sequences can provide insights to protein solubility. For example, the analysis of dipeptide scores shows high propensity of α-helix structure and thermophilic proteins to be soluble.

**Conclusions:**

The propensities of individual dipeptides to be soluble are varied for proteins under altered experimental conditions. For accurately predicting protein solubility using SCM, it is better to customize the score card of dipeptide propensities by using a training data set under the same specified experimental conditions. The proposed method SCM with solubility scores and dipeptide propensities can be easily applied to the protein function prediction problems that dipeptide composition features play an important role.

**Availability:**

The used datasets, source codes of SCM, and supplementary files are available at http://iclab.life.nctu.edu.tw/SCM/.

## Background

Many proteins are produced in the form of insoluble aggregation that is a major obstruct for a lot of experiments, and the misfolded aggregation is called inclusion body. Many proteins form inclusion bodies when overexpressed in *Escherichia coli *(*E. coli*). These insoluble proteins need be solubilized and refolded to obtain functional proteins [1]. Protein solubility defined as the concentration of soluble proteins varies widely, ranging from almost complete insolubility to values of several hundreds of milligrams per milliliter under given experimental conditions of pH, temperature, buffer concentration, and additives [2].

Protein solubility is a major concern when making biochemical experiments. Accordingly, researchers usually do their possible efforts to get the soluble forms of proteins by regulating experimental conditions, including culture temperature, co-expression with solubility-enhanced proteins, efficient vectors, and host strains. All about adjustments in experimental conditions that in order to get soluble proteins are still trial-and-error procedures. There is a significant need for highly consistent and accurate methods for predicting solubility of proteins from sequences [3].

Due to various extrinsic and intrinsic factors that influence protein solubility, it is difficult to develop an accurate and universal prediction method for estimating protein solubility and change upon point mutation. Generally, computational sequence-based prediction methods focus on the intrinsic determination of solubility for proteins overexpressed in *E. coli *at the normal growth temperature of 37°C. Numerous studies aim to investigate the features which correlate well with solubility for designing accurate prediction algorithms.

Many studies show that the amino acid sequence play a crucial role in determining solubility of expressed proteins. That is confirmed by experiments that point mutations in an expressed protein sequence could change the expressed solubility status under the same experimental conditions [1,4-6]. So it can be known clearly that the primary structure is related to the propensity of a protein to form inclusion body or not in some way.

Many researchers predict solubility of expressed proteins in *E. coli *from their primary structures. The first predictive model with a regression analysis [7] used a database of 81 proteins and 6 parameters, including turn forming residue fraction, charge average, cysteine fraction, hydrophilicity index, proline fraction, and molecular weight. Davis et al., [8] found that only two of six parameters in [7], the turn forming residues and the charge average, influenced the solubility of overexpressed proteins in *E. coli*. Idicula-Thomas and Balaji adopted a discriminant analysis using 170 proteins and found that the most important parameters are threonine, asparagine and tyrosine fraction, aliphatic index, and tripeptide and dipeptide composition [9]. Idicula-Thomas et al., [3] proposed a support vector machine (SVM) based learning algorithm to predict protein solubility by evaluating three feature sets. The best accuracy of 72% is obtained using the set of 446 features, consisting of 20 reduced αbet sets, 6 physicochemical properties, 20 residues, and 400 dipeptides where 8000 tripeptide-composition features have no improvement in prediction accuracy [3].

Smialowski et al., established a large dataset and proposed a two-layered predictor PROSO combining SVM and Naive Bayes classifiers [10]. Magnan et al., used a huge dataset of 17,408 protein sequences and developed a two-stage SVM classifier using SVM and Naive Bayes classifiers [11]. Diaz et al., employed logistic regression with 32 features which potentially correlate well with solubility and established a dataset of 212 proteins where the solubility status is confirmed by biological experiments [12]. Chan et al., [13] predicted solubility of expressed proteins using SVM with accuracy of 83.51% where the dataset of 726 protein sequences is the combination of 6 different fusion tags and 121 target proteins. Smialowski et al., proposed a two-layered method PROSO II using a primary Parzen model and a logistic regression classifier for protein solubility prediction [14].

The motivation of this study arises mainly from the following aspects: 1) the features of amino acid and dipeptide composition are useful for solubility prediction, but there are very few studies on estimating propensities of individual residues and dipeptides to be soluble; 2) it is also desirable to know the relationship between protein solubility and some biochemical and physicochemical properties of amino acid residues; 3) the existing SVM-based classifiers with a set of selected features have high generalization ability and prediction accuracy, but they suffer from low interpretability of insight to solubility; and 4) a simple and easily interpretable prediction method with an acceptable accuracy is more useful.

In this study, we propose a novel scoring card method (SCM) by using dipeptide composition only to estimate solubility scores for predicting protein solubility from sequences. SCM estimates and optimizes the propensities of 400 individual dipeptides to be soluble using statistic discrimination between soluble and insoluble proteins, and an intelligent genetic algorithm [15] by maximizing prediction accuracy, respectively. The solubility score of a protein can be simply determined by using a weighted sum of all propensity scores and dipeptide composition. To evaluate SCM by performance comparisons with existing SVM-based classifiers, four data sets with different sizes and variation degrees of experimental conditions were used.

By analyzing the relationship between the 531 physicochemical properties in the AAindex [16] and the estimated solubility scores of residues, we can get some insights to protein solubility. For example, the properties of the hydrophobicity group have a wide range of correlation coefficient (R value) in [-0.31, 0.35]. This scenario agrees with the inconsistence of literature reports about the propensity of hydrophobicity due to different experimental conditions [9]. The property with the largest value R = 0.83 is the distribution (i.e., percentages) of amino acid residues in the α-helices in thermophilic proteins. This finding agrees with the high propensity of α-helices structure and thermophilic proteins reported in literature [9].

The performance comparison results show that the proposed SCM is effective for predicting protein solubility, compared with existing state-of-the-art SVM-based methods. The SCM method has potential ability to generate various score cards of dipeptides to predict protein functions where the features of dipeptide and amino acid composition play an important role in the prediction, such as the prediction of carbohydrate-binding proteins [17]. There are numerous potential applications of SCM to protein function prediction problems such as crystallization [18], predictions of subcellular localization and nuclear receptors [19], virulent protein [20], protein structure class and ion channel [21,22], and gene expression level [23].

## Methods

### Data sets

In this study, we do our best effort to utilize four data sets with different sizes and variation degrees of experimental conditions for evaluating the proposed method SCM. The first data set Sd957 is established by the authors that the solubility status is confirmed by biological experiments, and the other three data sets were the same with the existing studies [11,13] and [14], for performance comparisons.

#### Data sets Sd957 and Sd726

Expressed proteins with solubility states were collected based on literature reports [11-13], which were all expressed at the normal growth temperature of 37°C. Only the proteins used in previous work that the solubility status is confirmed by biological experiments were considered in this data set. The dataset called Sd957 consists of 285 soluble proteins and 672 insoluble proteins, collected mainly from three parts.

In the first part, a combination of the keywords inclusion bodies, soluble, *E. coli*, and overexpression was used to search PubMed for identifying proteins which have been overexpressed in *E. coli *under the normal growth condition [9]. The second part comes from the dataset of 212 proteins, including 52 soluble proteins and 160 inclusion bodies [12]. The proteins in the two parts mentioned above have no fusion tags. The third part comes from the used dataset of 726 protein sequences in [13] (named Sd726) that the dataset is the combination of six different fusion tags and 121 target proteins. Different fusion tags combined with the same target protein may bring the expressed protein resulting in distinct status. There are 980 proteins after integration of the three parts. After filtering by deleting duplicate proteins, 957 proteins remain in the final dataset. The used dataset is available at http://iclab.life.nctu.edu.tw/SCM/.

#### Data set SOLproDB

This dataset SOLproDB with 17408 (8704 soluble and 8704 insoluble) proteins is presented in [11] that were collected from major protein databases such as Protein Data Bank (PDB), SwissProt, TargetDB and literature report [9]. Although the study [11] assumes that SOLproDB comes from the same experimental condition, the proteins from TargetDB possibly have ~20% of protein sequences which are expressed using different hosts. After removing protein sequences which contain unknown amino acid residues, this dataset comprises 16902 (8212 soluble and 8690 insoluble) proteins. Then, the comparison results between SCM and SOLproDB are obtained using this refined dataset.

#### Data set SdPROSOII

Smialowski et al., proposed a two-layered method PROSOII using a primary Parzen model and a logistic regression classifier for protein solubility prediction [14].

The data set SdPROSOII is used by [14] consisting of 82,299 proteins with 90% sequence identity. SdPROSOII is established by selecting proteins from the pepcDB and PDB databases. For performance comparison, the sequence identity of soluble and insoluble sets separately is further reduced at the sequence identity 25% as [14] using the CD-HIT program [24].

### The proposed method SCM

The proposed scoring card method SCM is an efficient and generalized method for creating various kinds of dipeptide scoring cards for predicting protein functions from whole sequences. The suitable prediction problems are those that the amino acid and dipeptide composition play an important role in serving as significantly effective features. The description of SCM is given in a general-purpose algorithm without using heuristics or specific domain knowledge. The SCM method can be applied to other prediction problem without significant modifications. Of course, the generic score matrix of dipeptides can be further customized and utilized with other complementary features for advancing prediction accuracy.

**Figure 1:**
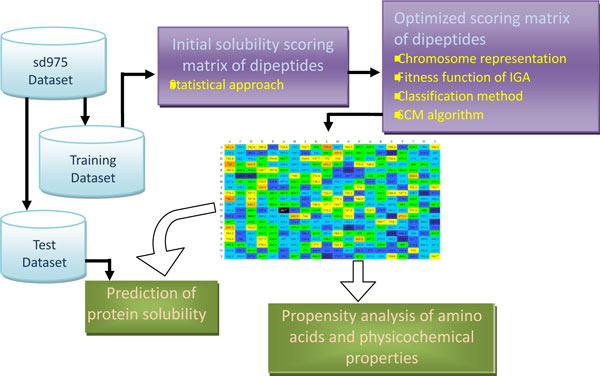
**The system flowchart of the proposed scoring matrix method**.

The system flowchart of the SCM method with propensity analysis is shown in Figure 1. The description of SCM consists of the following parts: 1) creation of data sets for both training and independent test, 2) establishment of an initial scoring matrix for propensity of dipeptides using a statistical approach, 3) optimized solubility scoring matrix of dipeptides, 4) prediction of protein solubility, and 5) propensity analysis of amino acids and physicochemical properties.

#### Creation of data sets

A 10-fold cross validation experiment is adopted to evaluate SCM for predicting protein solubility. For each specified data set, a scoring matrix of dipeptides is customized in the SCM method. The dataset sd957 with the solubility status confirmed by biological experiments is used to illustrate SCM and analyze the scoring matrix of dipeptides. The dataset sd957 is randomly divided into 766 training (219 soluble, 547 insoluble) and 191 test (50 soluble, 141 insoluble) proteins. The training data set is used for optimizing the solubility scoring matrix (SSM) and determining the suitable threshold value for classifying the query sequence as soluble or insoluble proteins.

#### Initial scoring matrix using a statistical approach

The solubility scoring matrix (SSM) of dipeptides consisting of 400 dipeptide scores is generated using a coarse-to-fine approach. The initial SSM is created by using a statistical approach based on the dipeptide composition and then the final SSM is optimized by using an intelligent genetic algorithm (IGA) [15]. The initial SSM is obtained using the following algorithm. The input is the two classes of soluble and insoluble sequences. The output is an initial SSM of dipeptides. The larger the solubility score of the dipeptide, the larger contribution to the propensity of a protein is to be soluble.

Step 1: Calculate the numbers of 400 dipeptides in each class. For example, the numbers of dipeptide AA in soluble and insoluble classes are 1067 and 1833, respectively.

Step 2: Normalize the dipeptide composition by dividing the numbers using the total numbers of dipeptides in each class. For example, the total numbers of dipeptides in soluble and insoluble classes are 97,147 and 217,263, respectively. Therefore, the compositions of AA are 0.01098 and 0.0084, respectively.

Step 3: The scores of SSM for an individual dipeptide are obtained by subtracting the score of the insoluble class from that of the soluble class. For example, the score of AA is 0.00258 (= 0.01098 - 0.0084).

Step 4: Normalize the scores of all dipeptides into the range [0, 1000]. The score of AA is 794.

The scores of dipeptides in SSM are highly correlated to the relative contribution of dipeptides to protein solubility prediction using SCM that is first presented in literature. To further quantify the relative contribution of each amino acid to protein solubility, we average the scores of dipeptides AX and XA where X can be any amino acid and assign the averaged score to the amino acid A. The SSM of amino acids can be therefore derived. If the amino acid composition (i.e., percentages) of a certain protein has a high correlation with the SSM of amino acids, this protein is easy to predict as a soluble protein.

#### Optimized solubility scoring matrix

The initial SSM is further optimized by using IGA, an efficient evolutionary algorithm for solving large parameter optimization problem. In this problem, 400 real-valued variables for encoding the dipeptide scores to be optimized. For applying IGA to parameter optimization problem, both the fitness function (or called objective function in optimization algorithm) and chromosome representation in which the parameters are encoded need to be specified. After designing the fitness function and chromosome representation, the IGA algorithm of SCM is also given, described below.

##### Fitness function and chromosome representation

The fitness function of SCM comprises two parts that concern both consistency and accuracy. To increase consistency, a Pearson's correlation coefficient (R value) between the optimized SSM and the initial one of amino acids should be maximized. This criterion is derived from the hypothesis that the initial SSM of amino acids has meaningful information and should be conserved provided that the training data set is sufficiently large with nearly the same experimental conditions.

To maximize the prediction accuracy, the area under the ROC curve (AUC) is an effective criterion originally used in Machine Learning to compare binary classification rules [25]. By varying the threshold value for classifying the sequences in the validation data set into soluble and insoluble classes, the ROC curve and the corresponding AUC can be calculated. After the best SSM with the largest AUC is obtained, the threshold value with the highest overall accuracy is selected. Finally, the fitness function of an SSM is defined by maximizing a weighted sum of AUC and R as follows:

(1) $$\text{Max}.\;\text{Fit}\left(\text{SSM}\right)\;=\;{\text{W}}_{1}\times \text{AUC}+{\text{W}}_{2}\times \text{R},$$

where W_1 _and W_2 _are user-defined weights for the multi-objective fitness function. In this work, W_1 _and W_2 _are set to 0.9 and 0.1, respectively, after evaluating other weight combinations (see Results section).

All the 400 real-valued variables are encoded into a chromosome of IGA where each variable belongs to the range [0, 1000]. For obtaining a high generalization ability of SSM for independent test, a 10-folds cross-validation assessment is utilized in evaluating the fitness function. IGA uses a divide-and-conquer strategy to solve large-scale optimization problems [15]. The detailed method can be referred to the work [15,26,27].

##### IGA algorithm of SCM

The initial SSM is obtained from the statistical method based on the training data set mentioned above. The IGA algorithm of SCM for obtaining an optimized SSM is described as follows:

Step 1: (Initialization) Randomly generate *Npop *individuals including the initial SSM. In this study, *Npop *= 40.

Step 2: (Evaluation) Compute fitness values of all individuals where *Ibest *is the best individual in the population.

Step 3: (Selection) Use a rank-based selection to select *Ps·Npop *individuals to establish a mating pool. In this study, *Ps *= 1.0.

Step 4: (Crossover) Perform the intelligence crossover operation [15] for each individual with *Ibest *to find the best two individuals among two parents and two children as the new children (the elitist strategy).

Step 5: (Mutation) Use a real-valued mutation operator to randomly mutate individuals with a mutation probability *Pm *(= 0.01). Mutation is not applied to *Ibest *to prevent the best fitness value from deteriorating.

Step 6: (Termination test) If a given termination condition is satisfied, stop this algorithm. Otherwise, go to Step 2. In this study, 20 generations are used as the stop condition.

Besides IGA, other efficient existing optimization algorithms are also available in achieving this goal of optimizing the SSM.

#### 4) Prediction of protein solubility

For a protein sequence P to be predicted, calculate the dipeptide composition first, named a set of *w_i _*belonging to [0,1], i = 1, ..., 400. Let *S*_i _be the corresponding score of SSM, i = 1, ..., 400. The score S of P is the weighted sum, defined as follows:

(2) $$S\left(\text{p}\right)=\sum_{i=1}^{400}w_{i}S_{i}.$$

If S(P) is greater than the given threshold value, P is classified as a soluble protein; otherwise, P is insoluble.

## Results and discussion

### Performance of solubility prediction using SCM

#### Effects of weights W_1 _and W_2_

The tested weight pairs (*W*_1_, *W*_2_) are (0.8, 0.2), (0.9, 0.1), and (1.0, 0) for evaluating SCM using the two representative data sets: Sd957 with similar experimental conditions and SOLproDB with diverse experimental conditions. The data set SOLproDB was also randomly divided into two data sets consisting of 8451 training (4106 soluble, 4345 insoluble) and 8451 test (4106 soluble, 4345 insoluble) sequences. To deal with the well-known undeterministic problem of genetic algorithms (GAs, i.e., the outcomes of GAs are not always the same due to the use of random numbers) [15], SCM was performed 10 independent runs on the training data sets of Sd957 and SOLproDB and the SSM with the highest accuracy is selected as the final optimized SSM.

The performance of SCM with different pairs of weights is shown in Table 1. According to Table 1, even though the value of *W***_2 _**was set to 0.1, the correlation coefficient R between the optimized SSM of amino acids and the initial SSM of amino acids is very high (> 0.9). Therefore, the weights (*W*_1_, *W*_2_) = (0.9, 0.1) are used in the following studies. The well-known SVM-based method with grid search for parameters C and γ is utilized as performance assessment where the libSVM software package was applied for all SVM experiments [28].

**Table 1:** The performance of SCM with different pairs of weights on two data sets Sd957 and SOLproDB.

Classifier (W_1_, W_2_)	Sd957			SOLproDB		
	
	Training (%)	Test (%)	R	Training (%)	Test (%)	R
SCM (0.8, 0.2)	83.52	82.72	0.981	59.99	58.99	1.000
SCM (0.9, 0.1)	84.47	84.29	0.953	59.99	58.99	1.000
SCM (1.0, 0)	84.99	87.43	0.682	63.85	60.00	0.776

R is a correlation coefficient.

#### Performance evaluation of SCM

The AUC, threshold value, training and test accuracies of SCM using an initial SSM on Sd957 with (*W*_1_, *W*_2_) = (0.9, 0.1) are 0.77, 403.13, 74.93%, and 74.87%, respectively. The optimized SSM was obtained using the initial SSM (shown in Table S1 [see Additional file 1]). The detailed results of 10 independent runs on Sd957 using an optimized SSM are given in Table 2. The SSM of Experiment 4 with the highest training accuracy was selected for future analysis. The AUC, threshold value, training and test accuracies of using an optimized SSM are 0.89, 463.79, 84.47% and 84.29%, respectively. The optimization procedure can advance the training and test accuracies 9.54% and 9.42%, respectively. In Table 3, the training and test accuracies of SVM on Sd957 are 85.38% and 84.29%, respectively. The test accuracy 84.29% of SVM is equal to that of SCM. The training accuracy of the proposed SCM and SVM methods are 59.99% and 65.35% on SOLproDB, respectively, while the test accuracies of these two methods are 58.99% and 62.49%, respectively (Table 3). The results reveal that the SCM and SVM methods using the same dipeptide composition features are comparable. However, the classification method of SCM is much simple and intuitive, compared with SVM.

**Table 2:** 10 independent runs of the scoring card method on Sd957.

**Exp**.	Fitness	Training (%)	Test (%)	Sensitivity	Specificity	AUC	R	Threshold
1	0.905	83.159	81.675	0.640	0.879	0.893	0.954	463.746
2	0.903	83.420	83.246	0.740	0.865	0.894	0.940	473.867
3	0.906	83.681	85.864	0.760	0.894	0.897	0.943	461.951
4	0.906	84.465	84.293	0.740	0.879	0.894	0.953	463.787
5	0.900	82.507	83.246	0.780	0.851	0.883	0.968	455.756
6	0.900	83.943	86.911	0.820	0.887	0.884	0.962	457.543
7	0.902	83.943	84.293	0.720	0.887	0.888	0.959	464.317
8	0.899	83.159	83.770	0.680	0.894	0.885	0.957	464.933
9	0.901	82.507	83.246	0.760	0.858	0.885	0.966	460.600
10	0.902	82.898	83.246	0.720	0.872	0.885	0.970	457.866
Mean	0.902	83.368	83.979	0.736	0.877	0.889	0.957	462.437
Std. Dev.	0.003	0.646	1.485	0.051	0.015	0.005	0.010	5.132

The experiment 4 having the best training accuracy is used for future analysis.

**Table 3:** Performance comparisons between SCM and SVM using the same dipeptide composition.

Classifier	Sd957		SOLproDB	
	
	Training (%)	Test (%)	Training (%)	Test (%)
SCM	84.47	84.29	59.99	58.99
SVM	85.38	84.29	65.35	62.49

**Figure 2:**
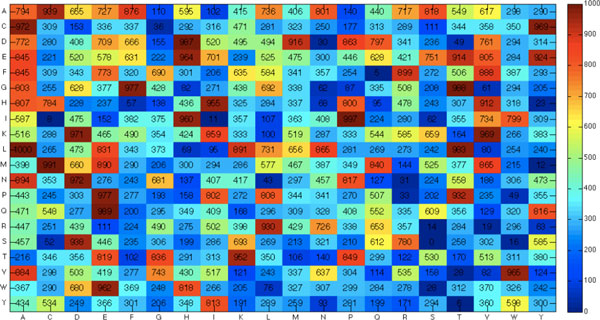
**Heat map of the optimized solubility scoring matrix of dipeptides**.

**Figure 3:**
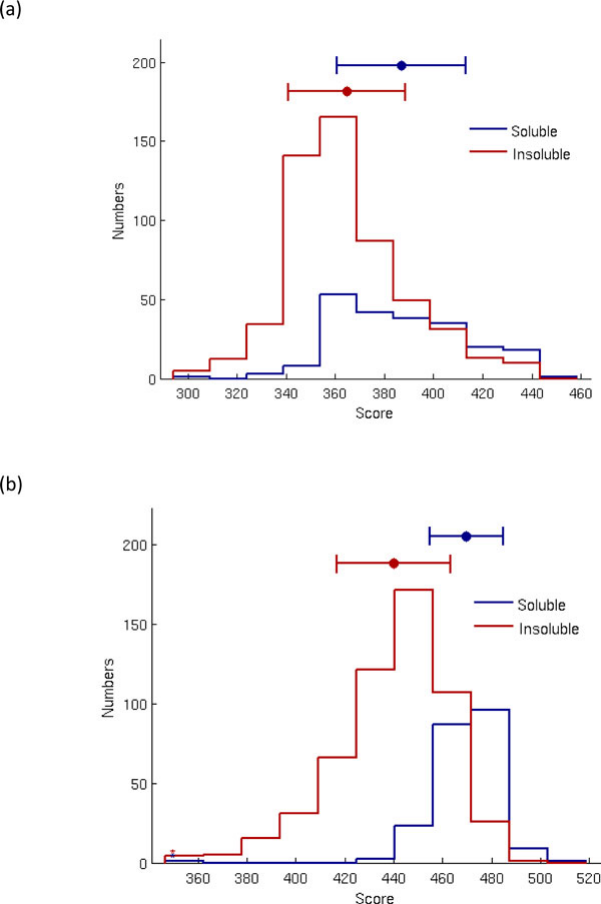
**The histogram of sequence solubility scores in the test data set**. (a) statistical SSM without optimization (b) optimized SSM.

The optimized solubility scoring matrix (SSM) of dipeptides obtained by the SCM method using sd957 is given in Figure 2, a heat map of the SSM of dipeptides. The three top-ranked dipeptides are LA, IP and MC with scores 1000, 997 and 991, respectively. The three dipeptides with the smallest scores are SS, FQ and YT with scores 0, 5 and 6, respectively. The histogram of sequence's solubility scores in the test data set is given in Figure 3. The range of most soluble proteins distributed is reduced after the optimization of IGA. Furthermore, the distributions for the soluble and insoluble data sets are more separable after optimization.

**Figure 4:**
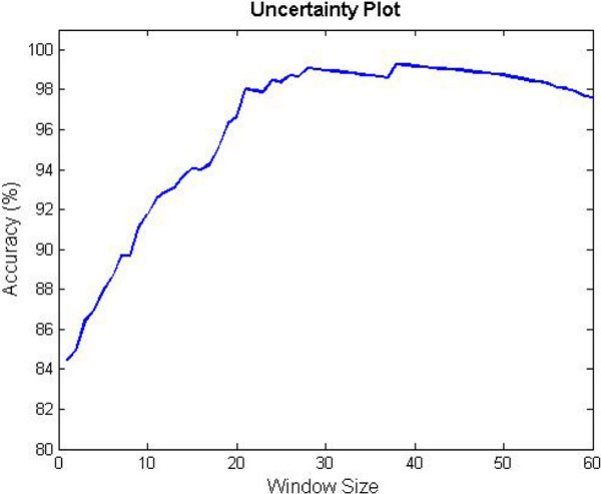
**The test accuracies for various sizes of uncertainty regions**.

The scoring card method classifies the query sequence based on the comparison between the score of the protein and the threshold value. We can extend the score range from the threshold value to form an uncertainty region. We can only make a decision of classification if the score of a query sequence does not belong to the uncertainty region. Figure 4 shows the test accuracies for various sizes of uncertainty regions. If the size of the uncertainty region is around 40 (i.e., uncertainty region is defined by 20 points distanced from best threshold score), the test accuracy is near to 99%. To advance the prediction accuracy, we can specify an adaptive uncertainty region and classify the sequence located in the uncertainty region using SVM with a number of complementary features.

### Comparing SCM with existing methods

#### Data set Sd726

To compare with the existing method implemented on the data set with similar experimental conditions [13], we implemented the same SCM method using the data set Sd726. The performance assessment is implemented in the similar way as the SVM-based method [13]. The data set was randomly divided into training and test data sets for 10 times. Each of the training data sets was independently used for optimizing SSMs. The results of SCM on Sd726 for the 10 independent runs are given in Table S2 [see Additional file 2]. In this experiment, SCM can achieve a mean test accuracy of 83.48%. On the other hand, the SVM-based method [13] using 617 features consisting of 84-nucleotide composition, 71 post-translational modifications, 400 dipeptides, and 62 nucleotide and protein features used in previous works achieved a test accuracy of 83.51%. The results reveal that the SCM method using the optimized SSM with dipeptide composition features only is comparable to the SVM-based method using a number of feature types.

#### Data sets SOLproDB and SdPROSOII

SOLpro is an SVM-based ensemble classifier (SVM on the first and Naïve Bayes on the second levels of classifier) using a combination of complementary sequence features described by Monomer frequencies, Dimer Frequencies, and Trimer Frequencies [11]. To investigate the generalization ability of the proposed SCM and the existing method SOLpro [11] across data sets with different experimental conditions, the cross-performance comparisons are conducted. The SCM predictor trained by Sd957 is evaluated using SOLproDB as an independent test data set. Similarly, the SOLpro predictor using Sd957 as an independent test data set. Unexpectedly, both experiments provided poor test accuracies (see Table 4). The predictors SOLpro and SCM can achieve only 49.21% and 53.90%, respectively. The 10-fold cross-validation accuracy of SOLpro using SOLproDB is 74.15% [11]. This unexpected result might be caused from the distinct experimental condition in both datasets. According to these results, the consistency of experimental conditions is considered as one of the major factors, which influences the correctness of solubility prediction in both SCM and SOLpro.

**Table 4:** The cross-performance comparison between SCM and SOLpro.

Classifier	Sd957	SOLproDB
SCM	84.29%	53.90%
SOLpro^1^	49.21%	74.15%^2^

^1^SOLpro classifier is downloaded from the SOLpro website http://solpro.proteomics.ics.uci.edu/.

^2^The 10-fold cross-validation accuracy of SOLpro using SOLproDB is obtained from the SOLpro publication [11].

SCM is also compared with the newly published method PROSO II [14] using the new data set SdPROSOII with sequence identity 25%. The 10-fold cross-validation accuracy of PROSO II is 69.9% obtained from [14] where SCM has a mean accuracy of 64.36%. Because the holdout data set in [14] is not available, we performed an independent test experiment by dividing SdPROSOII into two equal subsets for training and test. The training and test accuracies are 66.55% and 64.50%, respectively. Notably, the method PROSO II is a two-layered structure where the output of a primary Parzen window model and a logistic regression classifier serve as input of a second-level logistic regression classifier [14]. These results reveal the advantages of SCM, simplex, interpretability, and accurateness, compared to the much more complex method.

### Propensity analysis of dipeptides and amino acids

The SSM of dipeptides has shown its effectiveness in predicting protein solubility. The scores of dipeptides in SSM are highly correlated to the relative contribution of dipeptides to protein solubility. To further quantify the relative contribution of each amino acid to protein solubility, the initial SSM of amino acids is analyzed, given in Table 5. The four top-ranked residues have high propensity to be soluble and insoluble are {Ala, Glu, Asp, Lys} and {Trp, Cys, Gly, Ser} in order, respectively. The result is in good agreement with the study [7] based on the content of charged residues (Asp, Glu, Lys, and Arg).

**Table 5:** The initial solubility scoring matrix of amino acids.

Amino acid	Score	*P*_α_	KUMS000103 (%)
A-Ala	494.3	1.39	14.1
E-Glu	445.6	1.35	8.8
D-Asp	386.2	0.89	5.7
K-Lys	358.5	1.11	7.7
M-Met	333.6	1.21	3.3
L-Leu	357.3	1.32	9.1
F-Phe	320.6	1.01	5
V-Val	362.8	0.89	5.9
P-Pro	319.5	0.5	0.7
I-Ile	360.4	1.04	7.1
H-His	317.9	0.92	2
Q-Gln	326.1	1.29	3.7
R-Arg	347.1	1.17	5.5
T-Thr	333.0	0.76	4.4
N-Asn	311.4	0.77	3.2
Y-Tyr	293.7	0.95	4.5
S-Ser	265.4	0.82	3.9
G-Gly	313.4	0.47	4.1
C-Cys	303.0	0.74	0.1
W-Trp	306.5	1.06	1.2

The turn forming residues Asn, Gly, Pro and Ser having a high propensity to be insoluble favor inclusion body formation [7,8]. The amino acids Pro (P), Asn (N), Ser (S) and Gly (G) have scores ranked at 9, 15, 17 and 18 that agree with the propensity to be insoluble. It has been proved that insoluble proteins contain less negative charged amino acids (Glu and Asp) [29]. The amino acids Glu and Asp have scores ranked at 2 and 3, respectively (Table 5). The study [29] observed that insoluble proteins more frequently had fewer negatively charged residues.

The 10 top-ranked dipeptides and their scores are LA, IP, MC, QE, GT, DH, LT, PE, GF and CA, with scores 1000, 997, 991, 989, 988, 987, 983, 977, 977, and 972, respectively. The dipeptides with high scores play an important role in increasing solubility. For example, the dipeptide GF recognized by the study [30] has high relation to the Kinetics of degradation and oil solubility of ester prodrugs of a model dipeptide (Gly-Phe). The 20 amino acid values of the property KUMS000103 are given in Table 5, which are the percentages of amino acid residues in the α-helices in thermophilic proteins. The amino acid residues Leu (9.1%) and Ala (14.1%) are the two top-ranked ones having the highest percentages. The dipeptide LA has the highest score 1000. It agrees with the high propensity of α-helix structure and thermophilic proteins to be soluble [9]. Notably, the dipeptide with the smallest score (0) is SS where S is a turn forming residue favoring inclusion body formation [7,8].

### Propensity analysis of physicochemical properties

**Figure 5:**
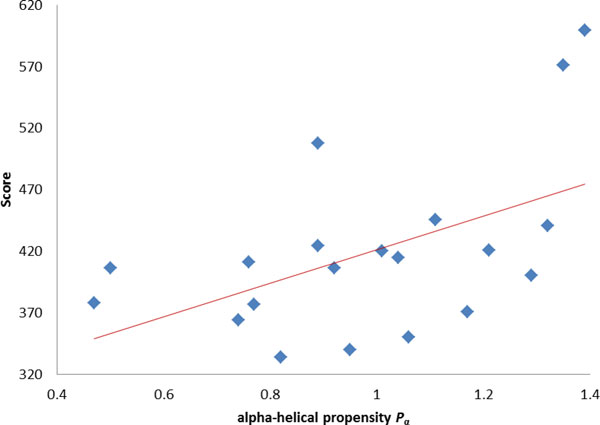
**The correlation coefficient R = 0.51 between the optimized SSM of amino acids and the α-helical propensity**.

It was deemed that the inclusion body proteins have more β-sheet and lesser α-helix structure [9,31]. The α-helical propensity *P*_α _to be the α-helix structure from [31] is given in Table 5. The correlation coefficient R is 0.58 between the initial SSM of amino acids and the α-helical propensity. The optimized SSM of amino acids is shown in Table S3 [see Additional file 3]. The correlation coefficient R = 0.51 between the optimized SSM of amino acids and the α-helical propensity is shown in Figure 5. The high correlation is in agreement with the high propensity of α-helix structure to be soluble and the effectiveness of SSM of amino acids.

To further investigated the propensity of physicochemical and biochemical properties, we analyzed all the 531 properties in the AAindex database [16]. Notably, the other 13 properties having the value 'NA' in a value set of amino acid index was discarded. Each property consists of a set of 20 numerical values for amino acids. The R values between some interesting physicochemical properties (a-helix, hydrophilicity, hydrophobicity, turn, charge, and thermophile) and the optimized SSM of amino acids are calculated. Some selected results of R values between interesting physicochemical properties and SSM of amino acids are shown in Table 6. If a high positive correlation exists between the SSM of amino acids and a specific property, the protein with this property is easy to predict as a soluble protein.

**Table 6:** Selected R values between some interesting physicochemical properties and the optimized SSM of amino acids.

Property	Keyword	**No**.	R (Max	Avg	Min	Var)
**α-helix**	**α-helix**	**39**	**(0.76**	**0.37**	**-0.42**	**0.12)**

R: 0.76	KUMS000103 Distribution of amino acid residues in the α-helices in thermophilic proteins					
0.54	PRAM900102 Relative frequency in α-helix					
-0.42	MUNV940102 Free energy in α-helical region					

Hydrophilicity	Hydrophilic	2	R (0.38	0.27	0.16	0.02)

R: 0.38	HOPT810101 Hydrophilicity value					
0.16	KUHL950101 Hydrophilicity scale					

Hydrophobicity	Hydrophobic	36	R (0.35	-0.09	-0.30	0.03)

R: 0.35	LEVM760101 Hydrophobic parameter					
-0.13	CIDH920103 Normalized hydrophobicity scales for α+β-proteins					
-0.30	CASG920101 Hydrophobicity scale from native protein structures					

Turn	Turn	26	R (0.38	-0.22	-0.57	0.06)

R: 0.38	OOBM850102 Optimized propensity to form reverse turn					
-0.19	PALJ810116 Normalized frequency of turn in α/β class					
-0.57	ROBB760108 Information measure for turn					

Charge	Charge	5	R (0.59	-0.01	-0.43	0.2)

R: 0.59	FAUJ880112 Negative charge					
-0.07	FAUJ880111 Positive charge					
-0.43	CHAM830108 A parameter of charge transfer donor capability					

Thermophile	thermophile	6	R (0.76	0.52	0.33	0.2)

R: 0.76	KUMS000103 Distribution of amino acid residues in the α-helices in thermophilic proteins					
0.56	FUKS010109 Entire chain composition of amino acids in intracellular proteins of thermophiles (percent)					
0.33	FUKS010105 Interior composition of amino acids in intracellular proteins of thermophiles (percent)					

The descriptions (definition) of AAindex ID are obtained from the AAindex database [16].

**Figure 6:**
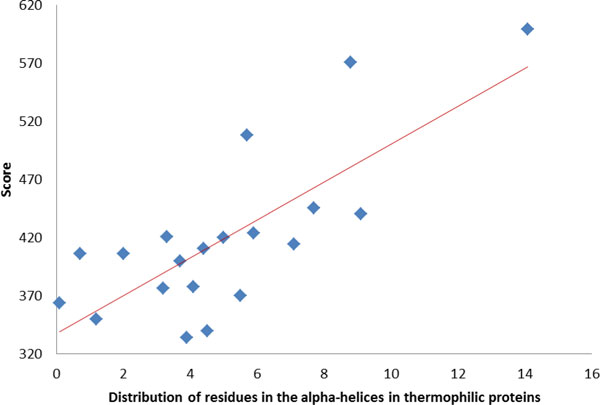
**The correlation coefficient R = 0.76 between the optimized SSM of amino acids and the property KUMS000103, the distribution of residues in the α-helices in thermophilic proteins**.

The property with the largest R value 0.76 shown in Table 6 is KUMS000103, the distribution (i.e., percentages) of amino acid residues in the α-helices in thermophilic proteins. The four top-ranked percentages of amino acids are 14.1% of Ala, 9.1% of Leu, 8.8% of Glu and 7.7% of Lys, where their propensity scores are at ranks 1, 6, 2 and 4, respectively. The weighted sum of using SSM of amino acids and amino acid composition would be relatively large. Therefore, these thermophilic proteins with α-helices are easy to predict as soluble proteins. The result agrees with the high propensity of α-helix structure and thermophilic proteins to be soluble [9]. Figure 6 shows the R = 0.76 between the optimized SSM of amino acids and the property KUMS000103, the distribution of residues in the α-helices in thermophilic proteins.

Table 6 gives the correlation values among the optimized SSM of amino acids and some interesting properties. From Table 6, the two group of α-helix and thermophilic properties have large mean values of R = 0.37 and 0.52, respectively. The properties of hydrophobicity group have a mean value of R = -0.09 and a wide range of R in [-0.31, 0.35]. This scenario agrees with the inconsistence of literature reports about the propensity of hydrophobicity due to different experimental conditions [9,32].

The aliphatic amino acids (including Ala, Ile, Leu, Pro and Val) are found that the appearance proportion is much higher in the thermophilic bacteria than other amino acids [33]. So they can be regarded as thermostability indicator of proteins. It is suggested that an increase in the thermostability of proteins might favor an increase in their solubility due to that solubility on overexpression and thermostability have a positive correlation [9]. The five aliphatic amino acids are the top-ten residues according to their scores (Table 5). The analysis results reveal that the SSMs of amino acids and dipeptides are informative and can be used to investigate the solubility and change upon point mutation.

### Distribution of top-ranked dipeptides on sequences

**Figure 7:**
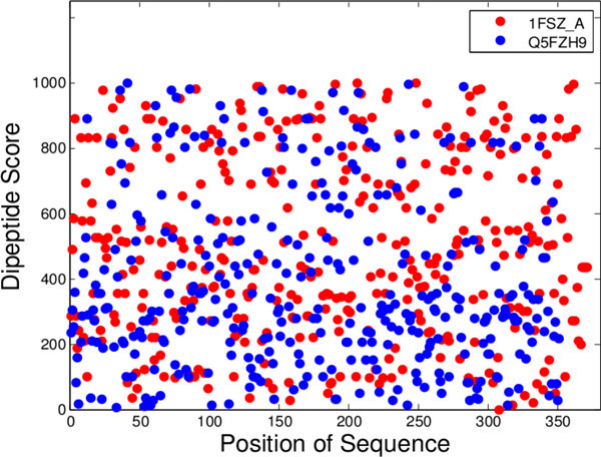
**Distribution of dipeptide scores on the positions of two typical sequences**. The protein 1FSZ_A with length 372 has a solubility score 499.92 predicted as a soluble protein, and Q5FZH9 with length 352 has a score 383.73 predicted as an insoluble protein where the threshold value is 463.79.

The proposed prediction method uses the weighted sum of dipeptide composition and SSM of dipeptides. To investigate the possibility that the top-ranked dipeptides tend to cluster in a certain region, we conducted an experiment for examining appearance (distribution) of location of dipeptides in protein sequences. Figure 7 shows the distribution of dipeptide scores on the positions of two typical sequences. One protein 1FSZ_A with length 372 and the other protein Q5FZH9 with length 352 were predicted as soluble and insoluble proteins, respectively. The result shows that both high-ranked and low-ranked dipeptides were uniformly distributed on the sequences. From this result, it might be observed that top-ranked dipeptides do not tend to cluster in a certain region and solubility is a global property of sequences for general proteins.

## Conclusions

This study has proposed a novel scoring card method (SCM) to estimate solubility scores of dipeptides and amino acid residues from a large dataset of sequences for predicting solubility of proteins and analyzing the propensity of physicochemical properties. The solubility scoring matrices (SSMs) of dipeptides and amino acids are easily manipulated. The classification method is very simple and the prediction result is easily interpretable. The SCM with SSMs performs well in predicting solubility, compared with existing complex methods using a large number of complementary features which correlate well with solubility. Furthermore, the propensity of physicochemical properties and the relative contribution to protein solubility are also analyzed by using the correlation value R. The results agreeing with the literature reports reveal that the SSMs are effective.

Since the solubility is influenced by various condition factors such as pH, temperature, buffer concentration, and various additives, the obtained SSM of dipeptides is only a generic matrix. If a customized SSM is needed, the datasets of protein solubility for specific expression conditions can be appended and the generic SSM can be tuned by using SCM. Since the proposed SCM method is effective for generating SSMs to predict protein solubility, the future work is to apply SCM to generate various kinds of scoring matrices of dipeptides for investigating protein function prediction problems where the features of dipeptide and amino acid composition play an important role.

## Competing interests

The authors declare that they have no competing interests.

## Authors' contributions

HLH designed the system, carried out the analysis, implemented programs, and participated in manuscript preparation. PC and TFK designed the system, implemented programs, and carried out the analysis. HCL, WLH, SJH, and LSS implemented programs and participated in designing the experiments. FLC and WLC provided biological Knowledge and analysis. SYH conceived and designed the experiments, supervised the project, and participated in writing the manuscript. All authors have read the final manuscript.

## Supplementary Material

Additional file 1 **Table S1**. The scores of the initial SSM using Sd957 (*.pdf) Click here for file

Additional file 2 **Table S2**. 10 independent runs of the scoring card method on Sd726 (*.pdf) Click here for file

Additional file 3 **Table S3**. The optimized solubility scoring matrix of amino acids (*.pdf) Click here for file
