# Supplementary material for: Prediction and analysis of protein solubility using a novel scoring card method with dipeptide composition
Source: BMC Bioinformatics. 2012 Dec 7;13(Suppl 17):S3. doi: 10.1186/1471-2105-13-S17-S3 (PMC3521471; doi:10.1186/1471-2105-13-S17-S3)
Supplement: Additional file 1 — Table S1. The scores of the initial SSM using Sd957 (*.pdf) [file 1471-2105-13-S17-S3-S1.pdf]

Table S1. The scores of the initial SSM using Sd957

	A	C	D	E	F	G	H	I	K	L	M	N	P	Q	R	S	T	V	W	Y
**A**	794	354	550	727	475	504	369	305	622	736	424	291	250	619	717	393	549	617	307	290
**C**	263	309	271	326	288	207	292	316	308	281	323	292	277	313	289	308	344	358	350	345
**D**	341	280	408	709	329	155	326	520	495	528	392	370	424	408	475	236	381	339	294	314
**E**	871	276	520	578	337	506	446	701	418	525	294	300	446	431	421	442	346	440	284	329
**F**	314	309	343	363	320	312	301	206	334	290	341	281	254	261	516	272	356	363	387	293
**G**	557	255	400	377	327	150	338	271	438	382	338	62	269	335	305	208	339	419	294	205
**H**	362	326	334	349	286	317	373	360	325	284	337	264	362	329	310	243	307	261	318	298
**I**	587	333	579	448	382	375	329	234	357	296	363	408	348	269	280	244	355	367	342	309
**K**	526	288	420	465	336	354	424	349	333	291	338	287	414	341	283	387	290	403	266	380
**L**	1000	265	473	598	343	373	271	350	346	314	345	381	281	269	273	242	314	217	254	240
**M**	326	352	369	362	290	206	300	294	286	396	348	387	349	332	346	328	377	392	304	301
**N**	613	353	285	276	243	130	286	407	417	340	297	457	340	261	176	224	254	327	306	244
**P**	443	305	303	460	277	391	297	303	272	313	344	314	270	295	280	202	474	235	292	323
**Q**	471	323	277	408	357	295	349	409	349	296	309	328	313	233	263	283	356	254	320	331
**R**	447	251	439	535	224	416	275	502	330	323	324	309	338	390	357	253	284	403	296	250
**S**	457	229	265	272	235	306	151	286	369	269	213	321	210	303	300	0	258	302	253	311
**T**	331	346	356	340	413	286	291	313	466	350	399	284	337	299	262	319	170	513	311	274
**V**	374	298	503	512	277	528	283	517	304	348	337	375	304	347	535	158	320	400	321	216
**W**	367	290	364	396	369	248	325	266	205	293	327	307	299	284	278	270	312	286	326	320
**Y**	434	318	266	411	301	206	345	301	191	289	305	203	302	199	273	294	272	360	304	300
