# Supplementary material for: Prediction and analysis of protein solubility using a novel scoring card method with dipeptide composition
Source: BMC Bioinformatics. 2012 Dec 7;13(Suppl 17):S3. doi: 10.1186/1471-2105-13-S17-S3 (PMC3521471; doi:10.1186/1471-2105-13-S17-S3)
Supplement: Additional file 2 — Table S2. 10 independent runs of the scoring card method on Sd726 (*.pdf) [file 1471-2105-13-S17-S3-S2.pdf]

Table S2. 10 independent runs of the scoring card method on Sd726

Exp.	Fitness	Train (%)	Test (%)	Sensitivity	Specificity	AUC	R	Threshold
1	0.919	84.709	83.333	0.783	0.857	0.913	0.970	451.597
2	0.918	85.933	85.933	0.697	0.935	0.912	0.977	450.736
3	0.920	85.168	83.333	0.739	0.878	0.914	0.973	464.915
4	0.921	85.015	85.015	0.654	0.942	0.915	0.974	456.741
5	0.912	83.486	83.333	0.652	0.918	0.905	0.978	449.592
6	0.916	84.557	86.111	0.652	0.959	0.908	0.984	453.463
7	0.920	85.321	81.944	0.565	0.939	0.915	0.960	454.259
8	0.918	85.168	81.944	0.696	0.878	0.912	0.977	457.896
9	0.921	85.168	80.556	0.652	0.878	0.916	0.970	457.747
10	0.920	84.098	83.333	0.609	0.939	0.914	0.975	454.408
Avg.	0.919	84.862	83.484	0.670	0.912	0.913	0.974	455.135
Sd.	0.003	0.688	1.784	0.062	0.036	0.004	0.006	4.464
