# Supplementary material for: Prediction and analysis of protein solubility using a novel scoring card method with dipeptide composition
Source: BMC Bioinformatics. 2012 Dec 7;13(Suppl 17):S3. doi: 10.1186/1471-2105-13-S17-S3 (PMC3521471; doi:10.1186/1471-2105-13-S17-S3)
Supplement: Additional file 3 — Table S3. The optimized solubility scoring matrix of amino acids (*.pdf) [file 1471-2105-13-S17-S3-S3.pdf]

Table S3. The optimized solubility scoring matrix of amino acids

Amino acid	Score
A-Ala	599.425
C-Cys	363.825
D-Asp	507.95
E-Glu	570.85
F-Phe	420.125
G-Gly	378.05
H-His	406.175
I-Ile	414.75
K-Lys	445.275
L-Leu	440.725
M-Met	420.875
N-Asn	376.65
P-Pro	406.225
G-Gln	400.025
R-Arg	370.575
S-Ser	334.1
T-Thr	411.025
V-Val	424.175
W-Trp	350.0
Y-Tyr	339.8
